# Synergistic Cues from Diverse Bacteria Enhance Multicellular Development in a Choanoflagellate

**DOI:** 10.1128/AEM.02920-19

**Published:** 2020-05-19

**Authors:** Ella V. Ireland, Arielle Woznica, Nicole King

**Affiliations:** a Department of Molecular and Cell Biology, University of California, Berkeley, Berkeley, California, USA; b Howard Hughes Medical Institute, University of California, Berkeley, Berkeley, California, USA; University of Illinois at Chicago

**Keywords:** EroS, RIF-1, *Salpingoeca rosetta*, choanoflagellate, chondroitinase, host microbe, multicellularity, outer membrane vesicles, rosette-inducing factor, sulfonolipid

## Abstract

Bacteria regulate the life histories of diverse eukaryotes, but relatively little is known about how eukaryotes interpret and respond to multiple bacterial cues encountered simultaneously. To explore how a eukaryote might respond to a combination of bioactive molecules from multiple bacteria, we treated the choanoflagellate Salpingoeca rosetta with two sets of bacterial cues, one that induces mating and another that induces multicellular development. We found that simultaneous exposure to both sets of cues enhanced multicellular development in *S. rosetta*, eliciting both larger multicellular colonies and an increase in the number of colonies. Thus, rather than conveying conflicting sets of information, these distinct bacterial cues synergize to augment multicellular development. This study demonstrates how a eukaryote can integrate and modulate its response to cues from diverse bacteria, underscoring the potential impact of complex microbial communities on eukaryotic life histories.

**IMPORTANCE** Eukaryotic biology is profoundly influenced by interactions with diverse environmental and host-associated bacteria. However, it is not well understood how eukaryotes interpret multiple bacterial cues encountered simultaneously. This question has been challenging to address because of the complexity of many eukaryotic model systems and their associated bacterial communities. Here, we studied a close relative of animals, the choanoflagellate *Salpingoeca rosetta*, to explore how eukaryotes respond to diverse bacterial cues. We found that a bacterial chondroitinase that induces mating on its own can also synergize with bacterial lipids that induce multicellular “rosette” development. When encountered together, these cues enhance rosette development, resulting in both the formation of larger rosettes and an increase in the number of rosettes compared to rosette development in the absence of the chondroitinase. These findings highlight how synergistic interactions among bacterial cues can influence the biology of eukaryotes.

## Abstract (precis)

Eukaryotic biology is profoundly influenced by interactions with diverse environmental and host-associated bacteria. However, it is not well understood how eukaryotes interpret multiple bacterial cues encountered simultaneously. This question has been challenging to address because of the complexity of many eukaryotic model systems and their associated bacterial communities. Here, we studied a close relative of animals, the choanoflagellate *Salpingoeca rosetta*, to explore how eukaryotes respond to diverse bacterial cues. We found that a bacterial chondroitinase that induces mating on its own can also synergize with bacterial lipids that induce multicellular “rosette” development. When encountered together, these cues enhance rosette development, resulting in both the formation of larger rosettes and an increase in the number of rosettes compared to rosette development in the absence of the chondroitinase. These findings highlight how synergistic interactions among bacterial cues can influence the biology of eukaryotes.

## INTRODUCTION

Eukaryotes, including animals and their closest living relatives, choanoflagellates, encounter abundant and diverse bacteria in the environment (1–3). However, interactions among eukaryotes and bacteria can be challenging to study in animal models due to the complex physiology of the hosts and the large number of oftentimes unculturable bacteria present, each of which releases diverse molecules (4–6). Multiple types of intestinal bacteria are required to induce full immune maturation in mice and humans, but it remains unclear whether this is due to interactions among the bacteria or the integration by the host of multiple independent bacterial cues (7–11). The interaction of a eukaryote with multiple partners can change the magnitude or directionality of each pairwise interaction (12), and it can be challenging to measure the functional and fitness effects of such complex networks (13). Therefore, simpler model systems may be necessary to investigate how animals and other eukaryotes integrate information from multiple bacterial cues encountered at the same time.

**FIG 1:**
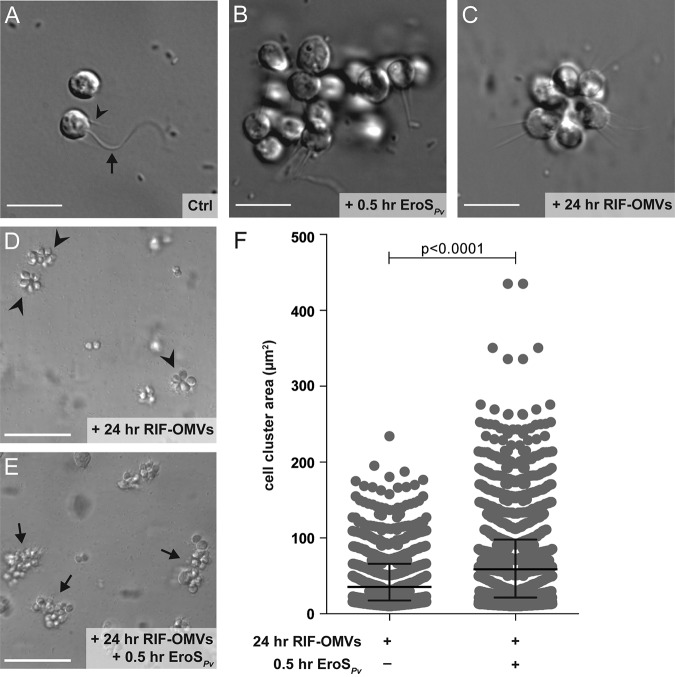
Rosettes swarm in response to the EroS*_Pv_* mating factor. (A to C) Bacterial cues regulate mating and multicellularity in *S. rosetta*. Bars, 10 μm. (A) *S. rosetta* grown in the presence of the prey bacterium *E. pacifica* (Ctrl) proliferated as solitary cells. This culture served as the foundation for all experiments in this study. A typical *S. rosetta* cell has an apical collar (arrowhead) surrounding a single flagellum (arrow). (B) *S. rosetta* formed mating swarms within 0.5 h of treatment with the bacterially produced chondroitinase EroS*_Pv_*. (C) *S. rosetta* solitary cells developed into rosettes through serial rounds of cell division within 24 h of treatment with RIF-OMVs from the bacterium *A. machipongonensis*. (D and E) Rosettes swarm in the presence, but not in the absence, of EroS*_Pv_*. Bars, 50 μm. (D) After 24 h of treatment with a 1:1,000 dilution of RIF-OMVs and BSA (carrier control), solitary cells in an SrEpac culture developed into rosettes (arrowheads) but did not swarm. (E) Swarms of rosettes (arrows) formed after 24 h of treatment with a 1:1,000 dilution of RIF-OMVs followed by 0.5 h of treatment with 0.05 U/ml (0.2 to 1 μg/ml, ∼2 to 8 nM) EroS*_Pv_*. (F) Scatterplot of the surface areas of cell clusters from SrEpac cultures treated with a 1:1,000 dilution of RIF-OMVs for 24 h followed by 0.5 h of incubation either with BSA (carrier control) or with 0.05 U/ml (0.2 to 1 μg/ml, ∼2 to 8 nM) EroS*_Pv_*. According to the approach described in reference 18, we generated a binary mask to measure cell cluster area (the area of each cell, rosette, or swarm) (see Fig. S1 in the supplemental material). EroS*_Pv_* treatment resulted in clusters of cells, including swarms of rosettes (median, 58.7 μm^2^; interquartile range, 21.6 to 98.0 μm^2^), whose areas were significantly larger than those measured in the rosette-only control (median, 35.5 μm^2^; interquartile range, 17.8 to 65.9 μm^2^) (Kolmogorov-Smirnov test). In total, 875 cell cluster areas from 3 biological replicates were plotted for the cultures treated with RIF-OMVs, and 1,359 cell cluster areas from 3 biological replicates were plotted for the cultures treated with RIF-OMVs plus EroS*_Pv_*.

The choanoflagellate Salpingoeca rosetta can serve as a simple model for studying interactions between bacteria and eukaryotes. Like all choanoflagellates, *S. rosetta* captures bacterial prey from the water column using an apical “collar complex” composed of a microvillar collar surrounding a single flagellum (Fig. 1A) (14, 15). In addition, like many animals (2, 16, 17), *S. rosetta* undergoes important life history transitions in response to distinct bacterial cues. For example, a secreted bacterial chondroitinase called EroS (for extracellular regulator of sex) produced by Vibrio fischeri, Proteus vulgaris, and select other *Gammaproteobacteria* induces solitary *S. rosetta* cells to gather into mating swarms (Fig. 1B) (18). The cells in mating swarms are not stably adherent and eventually resolve into pairs of cells that mate by undergoing cell and nuclear fusion, followed by meiotic recombination. When exposed to a different type of bacterial cue, specific sulfonolipids called rosette-inducing factors (RIFs) from the *Bacteroidetes* bacterium Algoriphagus machipongonensis, solitary cells of *S. rosetta* undergo serial rounds of cell division without separation, thereby resulting in the development of multicellular rosettes of cells (Fig. 1C) that are physically linked by cytoplasmic bridges and a shared extracellular matrix (19–22).

Mating and rosette development in *S. rosetta* differ in many respects, including the chemical nature of the bacterial cues (a protein versus lipids) and the underlying cell biology (cell aggregation versus incomplete cytokinesis). Moreover, the time scales of these processes differ, with mating swarms forming within 0.5 h of EroS treatment (18), while definitive rosettes require multiple rounds of cell division and are not observed until 11 to 24 h after exposure to RIFs (19–22).

Motivated by the existence of distinct *S. rosetta* life history transitions that can be regulated by biochemically unrelated bacterial cues, we used *S. rosetta* as a simple model for exploring how eukaryotes are influenced by environments filled with diverse bacterial cues. We investigated how *S. rosetta* responds to environments containing both the mating inducer EroS and the RIFs. We found that the initiation of mating behavior is unchanged in the presence of cues that induce rosette development. In contrast, rosette development is significantly enhanced by the presence of the mating inducer, revealing that *S. rosetta* integrates information from seemingly unrelated bacterial cues during rosette development.

## RESULTS

### Rosettes swarm in response to the EroS*_Pv_* mating factor.

In a culture containing *S. rosetta* and the prey bacterium Echinicola pacifica (together comprising a culture called SrEpac [23, 24]), solitary cells proliferated rapidly but underwent no other observable cell state transitions (Fig. 1A). When the SrEpac culture was treated with the secreted bacterial chondroitinase EroS from P. vulgaris (EroS*_Pv_*), *S. rosetta* cells formed mating swarms of 2 to 50 cells within 0.5 h (Fig. 1B; Table 1), as previously reported (18). In contrast, treatment of SrEpac with *A. machipongonensis* RIFs contained in outer membrane vesicles (RIF-OMVs) induced development of multicellular rosettes within 24 h (Fig. 1C and D; Table 1) (19, 22). RIF-OMVs were used for most experiments in this study, as they are stable and easily isolated and they fully recapitulate the inducing activity of live *A. machipongonensis* (22). Moreover, OMVs containing RIFs likely represent the most ecologically and physiologically relevant mode by which choanoflagellates encounter RIFs in the ocean (25). Because the precise concentrations of RIFs contained within OMVs are unknown, we instead used serial dilutions of RIF-OMV preparations to induce rosette development.

**TABLE 1:** *S. rosetta* phenotypes induced by EroS*_Pv_* and RIF-OMVs

Bacterial cue	Time (h) after induction	*S. rosetta* phenotype	Effect on swarming	Effect on rosette development
EroS*_Pv_*	0.5	Swarming	Induces	NA^*a*^
RIF-OMVs	0.5	Solitary	None	NA
EroS*_Pv_*+RIF-OMVs	0.5	Swarming	Induces	NA
EroS*_Pv_*	24	Swarming	Induces	None
RIF-OMVs	24	Rosette	None	Induces
EroS*_Pv_*+RIF-OMVs	24	Rosette plus swarming	Induces	Enhances

a NA, not applicable.

We then tested how mature rosettes (formed in response to pretreatment with RIF-OMVs for 24 h) would respond to the mating inducer EroS. After treatment with EroS*_Pv_* for 0.5 h, the preformed rosettes gathered into swarms that were quantifiable by their increase in area (median, 58.7 μm^2^; interquartile range, 21.6 to 98.0 μm^2^) compared to that of untreated rosettes (median, 35.5 μm^2^; interquartile range, 17.8 to 65.9 μm^2^) (Fig. 1D to F, Table 1). Therefore, rather than being mutually exclusive, the rosette morphology induced by RIF-OMVs and the swarming behavior induced by EroS*_Pv_* are compatible. This indicates that cells in a life history stage induced by one bacterial cue (in this case, RIF-OMVs) can respond to a second bacterial cue (EroS*_Pv_*). Swarms of choanoflagellate rosettes have not previously been reported, to our knowledge, and their ecological relevance is unknown.

### The mating inducer EroS*_Pv_* enhances rosette development.

We next investigated how single-celled *S. rosetta* in an SrEpac culture would respond to simultaneous exposure to EroS*_Pv_* and RIF-OMVs. SrEpac cultures treated solely with RIF-OMVs for 0.5 h, considerably less time than required for rosette development, did not produce swarms and were indistinguishable from untreated SrEpac cultures (Table 1; see also Fig. S1A to C in the supplemental material) (18, 19). Moreover, when SrEpac cultures were treated simultaneously with EroS*_Pv_* and RIF-OMVs for 0.5 h, the cells swarmed and the culture was indistinguishable from one treated with EroS*_Pv_* alone (Table 1; Fig. S1A, D, and E). Therefore, RIF-OMVs do not appear to influence the swarm-inducing activity of EroS*_Pv_* over time scales of 0.5 h or less.

**FIG 2:**
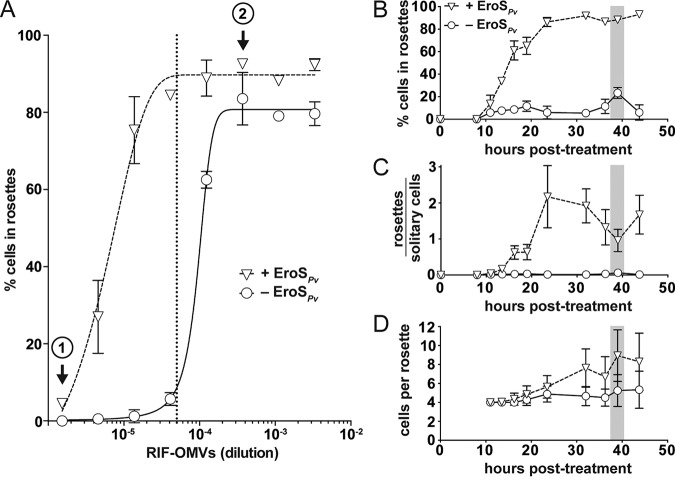
The mating inducer EroS*_Pv_* enhances rosette development. (A) EroS*_Pv_* enhances rosette induction by RIF-OMVs. Treatment of SrEpac with increasing concentrations of RIF-OMVs (circles) and BSA (carrier control) resulted in a concomitant increase in the percentage of cells in rosettes. Cotreatment of SrEpac with RIF-OMVs and 0.05 U/ml (0.2 to 1 μg/ml, ∼2 to 8 nM) EroS*_Pv_* (triangles) resulted in rosette development at concentrations of RIF-OMVs that did not otherwise induce rosettes (e.g., at circle 1). EroS*_Pv_* also increased the maximum percentage of cells in rosettes at saturating concentrations of RIF-OMVs (e.g., at circle 2). The 1:20,000 dilution of RIF-OMVs used for the sensitized rosette induction assays in panels B to D is indicated with a vertical dotted line. (B) Cotreatment of SrEpac with 0.05 U/ml (0.2 to 1 μg/ml, ∼2 to 8 nM) EroS*_Pv_* and a 1:20,000 dilution of RIF-OMVs leads to a dramatic increase in percentage of cells in rosettes throughout the course of rosette development relative to that in SrEpac treated only with RIF-OMVs and BSA (carrier control). After 39 h (shaded bar) of cotreatment with RIF-OMVs and EroS*_Pv_* (triangles), 88.2% ± 2.7% of *S. rosetta* cells were in rosettes, compared with 23.4% ± 4.9% of cells treated with RIF-OMVs alone (circles). (C) EroS*_Pv_* increased the ratio of rosettes to solitary cells in SrEpac cultures treated with RIF-OMVs. After 39 h (shaded bar) of cotreatment with a 1:20,000 dilution of RIF-OMVs and 0.05 U/ml (0.2 to 1 μg/ml, ∼2 to 8 nM) EroS*_Pv_* (triangles), the ratio of rosettes to solitary cells was 0.96 ± 0.31 compared with 0.06 ± 0.02 after treatment with RIF-OMVs and BSA (carrier control) (circles). (D) EroS*_Pv_* increased the number of cells per rosette in RIF-OMV-treated SrEpac cultures. After 39 h (shaded bar) of cotreatment with a 1:20,000 dilution of RIF-OMVs and 0.05 U/ml (0.2 to 1 μg/ml, ∼2 to 8 nM) EroS*_Pv_* (triangles), there were 8.9 ± 2.7 *S. rosetta* cells per rosette colony compared with 5.3 ± 1.7 cells per rosette colony after treatment with RIF-OMVs alone and BSA (carrier control) (circles). Means ± SDs from 3 biological replicates are plotted for each panel (A to D).

In contrast, when SrEpac cultures were cotreated with RIF-OMVs and EroS*_Pv_* for 24 h (long enough for rosettes to develop), the percentage of cells in rosettes increased markedly compared to that in cultures treated with RIF-OMVs alone (Fig. 2A; Table 1). Thus, EroS*_Pv_* enhances the rosette-inducing activity of RIF-OMVs. The enhancing activity of EroS*_Pv_* derived, in part, from the increased sensitivity of the culture to RIF-OMVs, allowing for rosette development at RIF-OMV concentrations that would otherwise fail to elicit rosette development. For example, at a nearly 10^−6^ dilution of RIF-OMVs, no rosettes were detected under the RIF-OMV-alone condition, while 4.5% ± 0.8% (mean ± standard deviation [SD]) of the cells in cultures cotreated with EroS*_Pv_* and RIF-OMVs were found in rosettes (Fig. 2A, circle number 1). In addition, when cells were exposed to saturating concentrations of RIF-OMVs (dilutions of ≥3.7 × 10^−4^), cotreatment with EroS*_Pv_* increased the percentage of cells in rosettes from a maximum of 83.6% ± 6.8% (mean ± SD) in cultures that were treated with RIF-OMVs alone to 92.6% ± 0.3% (mean ± SD) in cultures cotreated with RIF-OMVs and EroS*_Pv_* (Fig. 2A, circle number 2).

Enhancement of rosette development by the mating factor EroS was unexpected, and we next sought to understand the phenomenon in greater detail. To that end, we optimized a method for reproducibly inducing rosette development at low levels. Treating SrEpac with a 1:20,000 dilution of RIF-OMVs drove only a small percentage of cells (1% to 20%) into rosettes (Fig. 2A; see also Fig. S2A) and thereafter formed the basis of a “sensitized rosette induction assay” in which we could quantify the influence of EroS*_Pv_*. Under the conditions of the sensitized rosette induction assay, we found that EroS*_Pv_* enhanced rosette development in a concentration-dependent manner that saturated at 0.05 U/ml (0.2 to 1 μg/ml, ∼2 to 8 nM) (Fig. S2B). Using this sensitized rosette induction assay across a time series, the rosette-enhancing activity of EroS*_Pv_* at the population level became more evident (Fig. S2C). For example, while treatment of SrEpac with 1:20,000 RIF-OMVs yielded only 23.4% ± 4.9% (mean ± SD) of cells in rosettes at 39 h posttreatment, cotreatment with 1:20,000 RIF-OMVs and 0.05 U/ml (0.2 to 1 μg/ml, ∼2 to 8 nM) EroS*_Pv_* yielded 88.2% ± 2.7% (mean ± SD) of cells in rosettes (Fig. 2B).

These data demonstrated that cotreatment with EroS*_Pv_* increases the percentage of cells in rosettes at a population level but did not reveal whether EroS*_Pv_*-mediated enhancement works by (i) increasing the overall number of rosettes, (ii) increasing the average number of cells per rosette, or (iii) both. To test whether cotreatment with EroS*_Pv_* increased the number of rosettes formed, we induced SrEpac with either RIF-OMVs alone or RIF-OMVs plus EroS*_Pv_* and measured the ratio of rosette colonies to solitary cells. Cotreatment with RIF-OMVs and EroS*_Pv_* in the sensitized rosette induction assay consistently increased the ratio of rosette colonies to solitary cells throughout the time series. For example, at 39 h posttreatment, the ratio of rosettes to solitary cells after cotreatment with RIF-OMVs and EroS*_Pv_* was 0.96 ± 0.31 (mean ± SD) compared to 0.06 ± 0.02 (mean ± SD) after treatment with RIF-OMVs alone (Fig. 2C). The ratio of rosettes to solitary cells eventually plateaued, likely due to both solitary cells and rosettes (which can divide by fission [20]) dividing at the same rate. To test whether rosette size is influenced by cotreatment with EroS*_Pv_*, we used the sensitized rosette induction assay to compare the number of cells per rosette in cultures treated with RIF-OMVs alone to those treated with RIF-OMVs and EroS*_Pv_*. Cultures cotreated with EroS*_Pv_* formed larger rosettes (with 8.9 ± 2.7 [mean ± SD] cells per rosette colony at 39 h posttreatment) than those treated with RIF-OMVs alone (5.3 ± 1.7 [mean ± SD] cells per rosette colony at the same time point) (Fig. 2D). Importantly, cotreatment with EroS did not affect the change in cell density over time (Fig. S2D). Therefore, at limiting concentrations of RIF-OMVs, EroS*_Pv_* enhances the rosette-inducing activity of RIF-OMVs in at least two ways: at the population level, by increasing sensitivity to RIFs and the number of cells that initiate rosette development, and at the level of development, by increasing the maximal size of rosettes.

### Purified RIFs and EroS are sufficient for enhancement of rosette induction.

Because *A. machipongonensis* OMVs contain a suite of proteins, sugars, the sulfonolipid RIFs, and diverse other lipids, we next explored whether RIFs are sufficient for EroS*_Pv_*-mediated enhancement of rosette development or whether the phenomenon requires a non-RIF. For example, certain lysophosphatidylethanolamines (LPEs), lipids found alongside RIFs in *A. machipongonensis* OMVs, synergize with RIFs and enhance rosette induction, in part by increasing the resistance of larger rosettes to shear forces (22).

**FIG 3:**
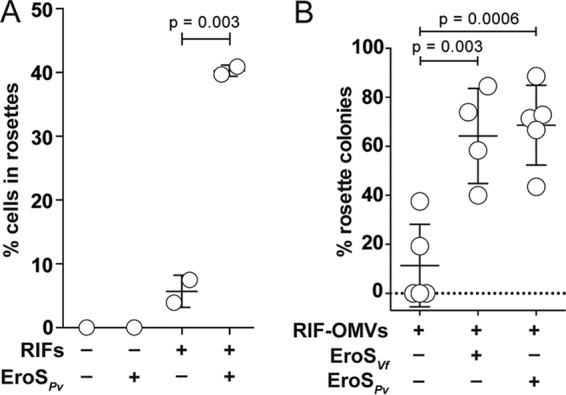
Purified RIFs and EroS are sufficient for enhancement of rosette induction. (A) Cotreatment of SrEpac with 10 μg/ml (16.7 μM) HPLC-purified RIFs and 0.05 U/ml (0.2 to 1 μg/ml, ∼2 to 8 nM) EroS*_Pv_* for 24 h resulted in an increase in the percentage of *S. rosetta* cells in rosettes compared to that after treatment with HPLC-purified RIFs and BSA (carrier control). Rosettes do not form in the absence of RIFs. Means ± SDs from 2 biological replicates are plotted (unpaired *t* test). (B) Cotreatment of SrEpac with a 1:20,000 dilution of RIF-OMVs and either 0.1% EroS from V. fischeri (EroS*_Vf_*) or 0.05 U/ml (0.2 to 1 μg/ml, ∼2 to 8 nM) EroS from P. vulgaris (EroS*_Pv_*) for 24 h resulted in an increase in the percentage of rosette colonies compared to that after treatment with RIF-OMVs and BSA (carrier control). Means ± SDs from 5 biological replicates (RIF-OMVs alone, RIF-OMVs plus EroS*_Pv_*) or 4 biological replicates (RIF-OMVs plus EroS*_Vf_*) are plotted (unpaired *t* test).

To test whether EroS*_Pv_* acts synergistically with RIFs or requires other components of RIF-OMVs, we compared rosette development in SrEpac cultures treated with high-performance liquid chromatography (HPLC)-purified RIFs (19, 22) with that in cultures cotreated with HPLC-purified RIFs and EroS*_Pv_*. Cotreatment with EroS*_Pv_* and purified RIFs caused a significant increase in the percentage of cells in rosettes compared to that with purified RIFs alone, indicating that the enhancement does not require other components of *A. machipongonensis* OMVs (Fig. 3A). Moreover, enhancement of rosette development was not restricted to P. vulgaris EroS. Cotreatment with purified V. fischeri EroS (EroS*_Vf_*) also significantly enhanced RIF-OMV-induced rosette development (Fig. 3B), revealing that the enhancing activity likely stems from the chondroitinase activity conserved between EroS*_Vf_* and EroS*_Pv_* rather than from a lineage-specific feature found only in EroS*_Pv_*. These findings show that simultaneous exposure to just two bacterial cues, RIFs and EroS, is sufficient to induce enhanced development of rosettes in *S. rosetta*.

## DISCUSSION

**FIG 4:**
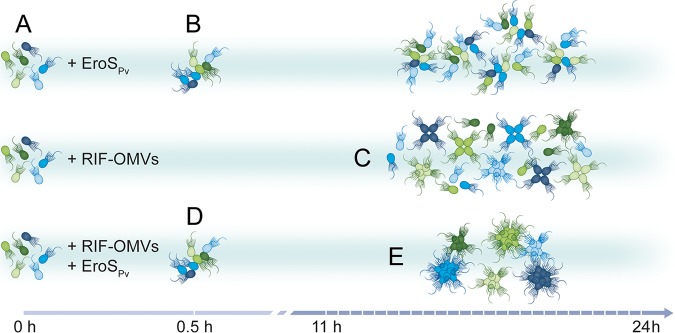
*S. rosetta* integration of bacterial cues. *S. rosetta* phenotypes induced over time by EroS*_Pv_*, RIF-OMVs, and the synergistic effect of both cues. (A) Untreated SrEpac proliferates as solitary cells. (B) Treatment with EroS*_Pv_* induces swarming of unrelated cells within 0.5 h. Cells with different genotypes (depicted as cells of different colors) can gather together to form nonclonal swarms. (C) Treatment with RIF-OMVs induces rosette development through cell division within 11 to 24 h. Cells in rosettes arise through serial rounds of cell division and share the same genotype (depicted as cells of the same color). (D) Cotreatment with RIF-OMVs and EroS*_Pv_* for 0.5 h results in swarming, showing that RIF-OMVs do not interfere with or enhance the activity of EroS*_Pv_*. (E) After 11 to 24 h of cotreatment with RIF-OMVs and EroS*_Pv_*, rosettes develop and swarm. Compared to that after treatment with RIF-OMVs alone, cotreatment with RIF-OMVs and EroS*_Pv_* induces the development of more rosettes and rosettes containing more cells.

We have shown here that the choanoflagellate *S. rosetta* can sense and respond to a mix of bacterial cues, each of which in isolation induces a distinct life history transition—mating or multicellularity. Together, these cues enhance multicellular development, increasing the number of cells in rosettes at a population level by increasing the proportion of rosettes to single cells and by increasing the number of cells per rosette (Fig. 2 and 4).

The *S. rosetta* targets for EroS and the sulfonolipid RIFs are as-yet unknown (18, 19), making it challenging to infer the specific mechanisms by which EroS might enhance rosette development. One possibility is that EroS may modify chondroitin sulfate proteoglycans through its chondroitinase activity, thereby improving access of RIF receptors to RIFs, potentially explaining the increased sensitivity of EroS-treated *S. rosetta* to RIF-OMVs (Fig. 2A). This type of mechanism would resemble the regulation of vascular endothelial growth factor receptor 2 (VEGFR2), whose activity is inhibited by *N*-glycosylation; enzymatic digestion of glycans on VEGFR2 enhances its response to the VEGF ligand (26).

In addition to increasing the sensitivity of *S. rosetta* to RIF-OMVs, EroS treatment also resulted in rosettes that contained more cells (Fig. 2D). A link between rosette size and extracellular matrix (ECM) modification was previously reported for another colony-forming choanoflagellate, Salpingoeca helianthica, in which treatment with a bovine chondroitinase resulted in a significantly increased number of cells per rosette (27). Furthermore, chemical perturbations of the *S. rosetta* ECM and computational modeling have shown that the material properties of the ECM, such as stiffness and volume, exert a physical constraint on rosette volume and morphology (28). Thus, EroS digestion of chondroitin sulfate in the *S. rosetta* ECM may relax these constraints and allow for increased proliferation of cells within rosettes.

Might *S. rosetta* in nature actually encounter the disparate types of bacteria that induce multicellularity and mating? Rosette development can be induced by diverse genera of marine bacteria, including *A. machipongonensis*, which was coisolated with *S. rosetta*, and Zobellia uliginosa, a macroalgal commensal (19, 29, 30). Likewise, mating can be induced by diverse *Vibrio* species (18), which are widespread in marine environments (31, 32). In both cases, the inducing factors are secreted, increasing the likelihood that *S. rosetta* could encounter both rosette-inducing and swarm-inducing cues in its environment. However, *S. rosetta* has only been isolated from the environment a single time (20), and little is known about its associated microbial communities. Although the ecological role of rosettes is unknown, they may have a fitness advantage in some environments. Previous studies have shown that *S. rosetta* colonies may draw in more water than single cells and form more food vacuoles, potentially indicating enhanced prey capture (33, 34). Another study detected no increase in prey capture and instead proposed that the larger size of rosettes may confer protection against predators (35).

Simple host-microbe interactions, in which a single bacterium elicits a clear phenotype from a eukaryotic host, have begun to reveal the molecular mechanisms by which bacteria influence the biology of eukaryotes. For example, V. fischeri colonizes and is sufficient to induce the development of the light organ in the bobtail squid, but this process only happens through the integration of multiple cues produced by V. fischeri—peptidoglycan and lipopolysaccharide (36). Likewise, we have previously shown that two types of molecules—sulfonolipid RIFs and specific LPEs—are necessary to recapitulate the rosette-inducing activity of live *A. machipongonensis* (22). Thus, interactions that are seemingly simple at the organismal level, for example, one bacterium and one eukaryote, can require complex interactions at the molecular level.

Given the underlying molecular complexity of interactions involving only one bacterium and one eukaryote, interactions among larger numbers of species are, perhaps unsurprisingly, complex and can yield a variety of outcomes, including synergistic effects (12). For example, arbuscular mycorrhizal fungi and rhizobia bacteria individually confer beneficial effects on plants, and the simultaneous presence of both groups in a tripartite association enhances these effects, increasing plant biomass to a greater extent than each partner could alone (37). Synergistic effects have also been demonstrated in interactions among eukaryotes and multiple bacterial species, such as in polymicrobial infections. Direct interactions among pathogens in polymicrobial infections (through metabolite exchange, signaling molecules, or direct contact) can synergistically increase the disease burden for the host (such as by increasing antibiotic resistance or virulence factor expression) (38). Eukaryotic integration of bacterial cues has also been observed in the mammalian immune system, in which immune receptors such as Toll-like receptors, T cell receptors, and coreceptors, each of which recognizes different bacterial ligands, synergize to enhance the response to multiple bacterial cues (39, 40).

Our finding that isolated cues from diverse environmental bacteria can synergize to enhance rosette development in *S. rosetta* (Fig. 3) demonstrates that this type of integration can occur at the level of the eukaryote, without requiring direct interactions among environmental bacteria. This is reminiscent of the tripartite association between the alga Ulva mutabilis and bacteria from *Cytophaga* and *Roseobacter*, in which secreted factors from both bacteria are required for complete algal morphogenesis (41, 42). Morphogenesis in the hydrozoan Hydractinia echinata is also regulated by synergistic bacterial cues: phospholipids and polysaccharides produced by distinct bacteria in biofilms (43).

In the future, identifying the *S. rosetta* target(s) of RIF and EroS activity will likely provide detailed insights into the molecular mechanisms underlying EroS-mediated enhancement of rosette development. The experimental tractability of *S. rosetta* and its susceptibility to the influences of environmental bacteria render it an exciting system in which to investigate the mechanisms by which eukaryotes grapple with a noisy and information-rich bacterial world.

## MATERIALS AND METHODS

### Choanoflagellate culturing conditions.

Artificial seawater (ASW) was prepared by diluting 32.9 g Tropic Marin sea salts in 1 liter of water for a salinity of 32 to 37 ppt (24). Seawater complete medium (SWC) was prepared by diluting 5 g/liter peptone, 3 g/liter yeast extract, and 3 ml/liter glycerol in ASW (24). SrEpac (Salpingoeca rosetta cocultured with the prey bacterium Echinicola pacifica, ATCC PRA-390 [24]) was cultured in 5% seawater complete medium (5% SWC [vol/vol] in ASW) at 22°C. Cultures were periodically frozen down and thawed, and several different thaws were used in this study. Cultures were passaged daily, 1 ml into 9 ml fresh medium in 25-cm^2^ cell culture flasks (Corning). Prior to rosette or swarm induction, cultures were diluted to 1 × 10^5^ choanoflagellate cells/ml in 5% SWC, and 100-μl volumes were aliquoted into a 96-well plate (Corning).

### Preparation of *A. machipongonensis* conditioned medium and isolation of RIF-OMVs.

Outer membrane vesicles were isolated from *A. machipongonensis* as described in reference 22. Briefly, *A. machipongonensis* (ATCC BAA-2233 [29]) was grown in 500 ml 100% SWC with shaking at 30°C for 48 h. The bacteria were pelleted, and the supernatant was filtered through a 0.2-μm filter to produce conditioned medium. Conditioned medium was then centrifuged at 36,000 × *g* for 3 h at 4°C (type 45 Ti rotor; Beckman Coulter). OMV-containing pellets were resuspended in 2 ml ASW.

### HPLC purification of RIFs.

RIFs were purified by HPLC as described in reference 22. Briefly, *A. machipongonensis* was grown in 20 liters marine broth medium (40.1 g/liter, CP.73; Carl Roth) with shaking at 30°C for 48 h. The cells were harvested by centrifugation and extracted with CHCl_3_/methanol (MeOH) (2:1, 4 liters). The organic extract was filtered and concentrated to give approximately 3 g crude lipid extract. The crude extract was dissolved in 60% MeOH (plus 0.1% NH_4_OH) and fractionated using C_18_ solid phase extraction (SPE) using a 10% step gradient of MeOH (60% to 100% MeOH plus 0.1 NH_4_OH). The resulting SPE fractions were analyzed for sulfonolipid-specific signals using liquid chromatography-mass spectrometry (LC-MS) and ^1^H nuclear magnetic resonance (^1^H-NMR). The fraction containing the RIF mix (RIF-1 and RIF-2) eluted with 90% MeOH (plus 0.1% NH_4_OH) during the SPE purification.

### Rosette induction.

Unless otherwise noted, SrEpac cultures were treated with a 1:1,000 dilution of RIF-OMVs and incubated for 24 h before imaging or counting. To induce a low level of rosette development in the sensitized rosette induction assay (Fig. 2B to D and 3B; see also Fig. S2B to D in the supplemental material), SrEpac cultures were treated with a 1:20,000 dilution of RIF-OMVs. HPLC-purified RIFs were resuspended in dimethyl sulfoxide (DMSO) and added at 10 μg/ml (16.7 μM) (Fig. 3A). DMSO was used as a carrier control for samples that did not receive HPLC-purified RIFs.

### Swarm induction.

Unless otherwise noted, cultures were treated with 0.05 U/ml (0.2 to 1 μg/ml, ∼2 to 8 nM) chondroitinase ABC from P. vulgaris (Sigma), referred to as EroS*_Pv_*; 0.1 mg/ml bovine serum albumin (BSA; Sigma) was used to resuspend EroS*_Pv_* and was used as a carrier control for samples that did not receive EroS*_Pv_*. EroS from V. fischeri (EroS*_Vf_*) (Fig. 3B) was purified as described in reference 18. Briefly, V. fischeri ES114 (ATCC 700601) was grown in 8 liters 100% SWC with shaking at 20°C for 30 h. The bacteria were pelleted, and the supernatant was filtered through a 0.2-μm filter, concentrated to 120 ml using a using a tangential flow filtration device with a 30 kDa Centramate filter (Pall OS030T12), and then ammonium sulfate precipitated and further separated by size exclusion chromatography. EroS*_Vf_* was added to SrEpac cultures at a final dilution of 0.1%.

### Rosette quantification.

To quantify the percentage of cells in rosettes, cultures were fixed with 1% formaldehyde, vortexed, mounted on a Bright-Line hemacytometer (Hausser Scientific), and counted on a Leica DMI6000B inverted compound microscope. Rosettes were defined as groups of four or more cells and were distinguished from swarms based on their resistance to mechanical shear and their stereotypical orientation, with their basal poles pointed inwards and their flagella out (20, 23). The numbers of solitary cells and rosettes, as well as the number of cells in each rosette, were counted for 3 biological replicates, until at least 200 cells were scored (per biological replicate). *P* values were calculated using an unpaired *t* test in Prism software (GraphPad).

### Swarm quantification.

Cell cluster areas were quantified as described in reference 18. Briefly, samples were imaged in 96-well glass-bottomed plates (Ibidi 89621) at ×10 magnification using transmitted light (bright field) on a Zeiss Axio Observer.Z1/7 widefield microscope with a Hamamatsu Orca-Flash 4.0 LT CMOS digital camera. Images from 3 biological replicates were processed and analyzed with the following functions in ImageJ: “smooth” to reduce bacterial background, “find edges” to further highlight choanoflagellate cells, “make binary” to convert to black and white, “close-” to fill in small holes, and “analyze particles” to calculate the area of each cell cluster. Particles smaller than 10 μm^2^ were removed to reduce background bacterial signal. *P* values were calculated using a nonparametric Kolmogorov-Smirnov test in Prism software (GraphPad).

## Supplementary Material

Supplemental file 1

## References

[1] McFall-Ngai MJ 2002 Unseen forces: the influence of bacteria on animal development. Dev Biol 242:1–14. doi:10.1006/dbio.2001.0522. 11795936

[2] Woznica A, King N 2018 Lessons from simple marine models on the bacterial regulation of eukaryotic development. Curr Opin Microbiol 43:108–116. doi:10.1016/j.mib.2017.12.013. 29331767 PMC6051772

[3] McFall-Ngai M, Hadfield MG, Bosch TCG, Carey HV, Domazet-Lošo T, Douglas AE, Dubilier N, Eberl G, Fukami T, Gilbert SF, Hentschel U, King N, Kjelleberg S, Knoll AH, Kremer N, Mazmanian SK, Metcalf JL, Nealson K, Pierce NE, Rawls JF, Reid A, Ruby EG, Rumpho M, Sanders JG, Tautz D, Wernegreen JJ 2013 Animals in a bacterial world, a new imperative for the life sciences. Proc Natl Acad Sci U S A 110:3229–3236. doi:10.1073/pnas.1218525110. 23391737 PMC3587249

[4] Eckburg PB, Bik EM, Bernstein CN, Purdom E, Dethlefsen L, Sargent M, Gill SR, Nelson KE, Relman DA 2005 Diversity of the human intestinal microbial flora. Science 308:1635–1638. doi:10.1126/science.1110591. 15831718 PMC1395357

[5] Lozupone CA, Stombaugh JI, Gordon JI, Jansson JK, Knight R 2012 Diversity, stability and resilience of the human gut microbiota. Nature 489:220–230. doi:10.1038/nature11550. 22972295 PMC3577372

[6] Donia MS, Fischbach MA 2015 Small molecules from the human microbiota. Science 349:1254766–1254766. doi:10.1126/science.1254766. 26206939 PMC4641445

[7] Tanoue T, Morita S, Plichta DR, Skelly AN, Suda W, Sugiura Y, Narushima S, Vlamakis H, Motoo I, Sugita K, Shiota A, Takeshita K, Yasuma-Mitobe K, Riethmacher D, Kaisho T, Norman JM, Mucida D, Suematsu M, Yaguchi T, Bucci V, Inoue T, Kawakami Y, Olle B, Roberts B, Hattori M, Xavier RJ, Atarashi K, Honda K 2019 A defined commensal consortium elicits CD8 T cells and anti-cancer immunity. Nature 565:600–605. doi:10.1038/s41586-019-0878-z. 30675064

[8] Atarashi K, Tanoue T, Oshima K, Suda W, Nagano Y, Nishikawa H, Fukuda S, Saito T, Narushima S, Hase K, Kim S, Fritz JV, Wilmes P, Ueha S, Matsushima K, Ohno H, Olle B, Sakaguchi S, Taniguchi T, Morita H, Hattori M, Honda K 2013 T_reg_ induction by a rationally selected mixture of *Clostridia* strains from the human microbiota. Nature 500:232–236. doi:10.1038/nature12331. 23842501

[9] Bouskra D, Brézillon C, Bérard M, Werts C, Varona R, Boneca IG, Eberl G 2008 Lymphoid tissue genesis induced by commensals through NOD1 regulates intestinal homeostasis. Nature 456:507–510. doi:10.1038/nature07450. 18987631

[10] Martin R, Nauta AJ, Ben Amor K, Knippels LMJ, Knol J, Garssen J 2010 Early life: gut microbiota and immune development in infancy. Benef Microbes 1:367–382. doi:10.3920/BM2010.0027. 21831776

[11] Chung H, Pamp SJ, Hill JA, Surana NK, Edelman SM, Troy EB, Reading NC, Villablanca EJ, Wang S, Mora JR, Umesaki Y, Mathis D, Benoist C, Relman DA, Kasper DL 2012 Gut immune maturation depends on colonization with a host-specific microbiota. Cell 149:1578–1593. doi:10.1016/j.cell.2012.04.037. 22726443 PMC3442780

[12] Afkhami ME, Rudgers JA, Stachowicz JJ 2014 Multiple mutualist effects: conflict and synergy in multispecies mutualisms. Ecology 95:833–844. doi:10.1890/13-1010.1. 24933804

[13] Mushegian AA, Ebert D 2016 Rethinking “mutualism” in diverse host-symbiont communities. Bioessays 38:100–108. doi:10.1002/bies.201500074. 26568407

[14] Dayel MJ, King N 2014 Prey capture and phagocytosis in the choanoflagellate *Salpingoeca rosetta*. PLoS One 9:e95577. doi:10.1371/journal.pone.0095577. 24806026 PMC4012994

[15] Leadbeater BSC 2015 The collared flagellate: functional morphology and ultrastructure, p 18–43. The choanoflagellates. Cambridge University Press, Cambridge, United Kingdom.

[16] Tebben J, Tapiolas DM, Motti CA, Abrego D, Negri AP, Blackall LL, Steinberg PD, Harder T 2011 Induction of larval metamorphosis of the coral *Acropora millepora* by tetrabromopyrrole isolated from a *Pseudoalteromonas* bacterium. PLoS One 6:e19082. doi:10.1371/journal.pone.0019082. 21559509 PMC3084748

[17] Shikuma NJ, Pilhofer M, Weiss GL, Hadfield MG, Jensen GJ, Newman DK 2014 Marine tubeworm metamorphosis induced by arrays of bacterial phage tail-like structures. Science 343:529–533. doi:10.1126/science.1246794. 24407482 PMC4949041

[18] Woznica A, Gerdt JP, Hulett RE, Clardy J, King N 2017 Mating in the closest living relatives of animals is induced by a bacterial chondroitinase. Cell 170:1175–1179. doi:10.1016/j.cell.2017.08.005. 28867285 PMC5599222

[19] Alegado RA, Brown LW, Cao S, Dermenjian RK, Zuzow R, Fairclough SR, Clardy J, King N 2012 A bacterial sulfonolipid triggers multicellular development in the closest living relatives of animals. Elife 1:e00013. doi:10.7554/eLife.00013. 23066504 PMC3463246

[20] Dayel MJ, Alegado RA, Fairclough SR, Levin TC, Nichols SA, McDonald K, King N 2011 Cell differentiation and morphogenesis in the colony-forming choanoflagellate *Salpingoeca rosetta*. Dev Biol 357:73–82. doi:10.1016/j.ydbio.2011.06.003. 21699890 PMC3156392

[21] Fairclough SR, Dayel MJ, King N 2010 Multicellular development in a choanoflagellate. Curr Biol 20:R875–R876. doi:10.1016/j.cub.2010.09.014. 20971426 PMC2978077

[22] Woznica A, Cantley AM, Beemelmanns C, Freinkman E, Clardy J, King N 2016 Bacterial lipids activate, synergize, and inhibit a developmental switch in choanoflagellates. Proc Natl Acad Sci U S A 113:7894–7899. doi:10.1073/pnas.1605015113. 27354530 PMC4948368

[23] Levin TC, Greaney AJ, Wetzel L, King N 2014 The *rosetteless* gene controls development in the choanoflagellate *S. rosetta*. Elife 3:e04070. doi:10.7554/eLife.04070. PMC4381721 25299189

[24] Levin TC, King N 2013 Evidence for sex and recombination in the choanoflagellate *Salpingoeca rosetta*. Curr Biol 23:2176–2180. doi:10.1016/j.cub.2013.08.061. 24139741 PMC3909816

[25] Lynch JB, Alegado RA 2017 Spheres of hope, packets of doom: the good and bad of outer membrane vesicles in interspecies and ecological dynamics. J Bacteriol 199:e00012-17. doi:10.1128/JB.00012-17. 28416709 PMC5512217

[26] Chandler KB, Leon DR, Kuang J, Meyer RD, Rahimi N, Costello CE 2019 *N*-Glycosylation regulates ligand-dependent activation and signaling of vascular endothelial growth factor receptor 2 (VEGFR2). J Biol Chem 294:13117–13130. doi:10.1074/jbc.RA119.008643. 31308178 PMC6721943

[27] Richter DJ, Fozouni P, Eisen MB, King N 2018 Gene family innovation, conservation and loss on the animal stem lineage. Elife 7:e34226. doi:10.7554/eLife.34226. 29848444 PMC6040629

[28] Larson BT, Ruiz-Herrero T, Lee S, Kumar S, Mahadevan L, King N 2020 Biophysical principles of choanoflagellate self-organization. Proc Natl Acad Sci U S A 117:1303–1311. doi:10.1073/pnas.1909447117. 31896587 PMC6983409

[29] Alegado RA, Grabenstatter JD, Zuzow R, Morris A, Huang SY, Summons RE, King N 2013 *Algoriphagus machipongonensis* sp. nov., co-isolated with a colonial choanoflagellate. Int J Syst Evol Microbiol 63:163–168. doi:10.1099/ijs.0.038646-0. 22368173 PMC3709532

[30] Matsuo Y, Suzuki M, Kasai H, Shizuri Y, Harayama S 2003 Isolation and phylogenetic characterization of bacteria capable of inducing differentiation in the green alga *Monostroma oxyspermum*. Environ Microbiol 5:25–35. doi:10.1046/j.1462-2920.2003.00382.x. 12542710

[31] Grimes DJ, Johnson CN, Dillon KS, Flowers AR, Noriea NF, Berutti T 2009 What genomic sequence information has revealed about *Vibrio* ecology in the ocean–a review. Microb Ecol 58:447–460. doi:10.1007/s00248-009-9578-9. 19727929

[32] Takemura AF, Chien DM, Polz MF 2014 Associations and dynamics of *Vibrionaceae* in the environment, from the genus to the population level. Front Microbiol 5:38. doi:10.3389/fmicb.2014.00038. 24575082 PMC3920100

[33] Roper M, Dayel MJ, Pepper RE, Koehl M 2013 Cooperatively generated stresslet flows supply fresh fluid to multicellular choanoflagellate colonies. Phys Rev Lett 110:228104. doi:10.1103/PhysRevLett.110.228104. 23767751

[34] L’Etoile NJ, King-Smith C 2020 Rosette colonies of choanoflagellates (*Salpingoeca rosetta*) show increased food vacuole formation compared with single swimming cells. J Eukaryot Microbiol 67:263–267. doi:10.1111/jeu.12780. 31872522

[35] Kirkegaard JB, Goldstein RE 2016 Filter-feeding, near-field flows, and the morphologies of colonial choanoflagellates. Phys Rev E 94:e052401. doi:10.1103/PhysRevE.94.052401. PMC6054299 27967109

[36] Koropatnick TA, Engle JT, Apicella MA, Stabb EV, Goldman WE, McFall-Ngai MJ 2004 Microbial factor-mediated development in a host-bacterial mutualism. Science 306:1186–1188. doi:10.1126/science.1102218. 15539604

[37] van der Heijden MG, De Bruin S, Luckerhoff L, van Logtestijn RS, Schlaeppi K 2016 A widespread plant-fungal-bacterial symbiosis promotes plant biodiversity, plant nutrition and seedling recruitment. ISME J 10:389–399. doi:10.1038/ismej.2015.120. 26172208 PMC4737930

[38] Murray JL, Connell JL, Stacy A, Turner KH, Whiteley M 2014 Mechanisms of synergy in polymicrobial infections. J Microbiol 52:188–199. doi:10.1007/s12275-014-4067-3. 24585050 PMC7090983

[39] Trinchieri G, Sher A 2007 Cooperation of Toll-like receptor signals in innate immune defence. Nat Rev Immunol 7:179–190. doi:10.1038/nri2038. 17318230

[40] Kroczek RA, Mages HW, Hutloff A 2004 Emerging paradigms of T-cell co-stimulation. Curr Opin Immunol 16:321–327. doi:10.1016/j.coi.2004.03.002. 15134781

[41] Spoerner M, Wichard T, Bachhuber T, Stratmann J, Oertel W 2012 Growth and thallus morphogenesis of *Ulva mutabilis* (Chlorophyta) depends on a combination of two bacterial species excreting regulatory factors. J Phycol 48:1433–1447. doi:10.1111/j.1529-8817.2012.01231.x. 27009994

[42] Grueneberg J, Engelen AH, Costa R, Wichard T 2016 Macroalgal morphogenesis induced by waterborne compounds and bacteria in coastal seawater. PLoS One 11:e0146307. doi:10.1371/journal.pone.0146307. 26745366 PMC4720170

[43] Guo H, Rischer M, Westermann M, Beemelmanns C 2019 Two distinct bacterial biofilm components trigger metamorphosis in the colonial hydrozoan *Hydractinia echinata*. bioRxiv doi:10.1101/2019.12.23.887182. PMC8262903 34154406

