# Supplementary material for: Synergistic Cues from Diverse Bacteria Enhance Multicellular Development in a Choanoflagellate
Source: Appl Environ Microbiol. 2020 May 19;86(11):e02920-19. doi: 10.1128/AEM.02920-19 (PMC7237790; doi:10.1128/AEM.02920-19)
Supplement: Supplemental file 1 [file AEM.02920-19-s0001.pdf]

**SUPPLEMENTAL MATERIAL**

# Synergistic cues from diverse bacteria enhance multicellular development in a choanoflagellate

**Ella V. Ireland[a], Arielle Woznica[a,*] and Nicole King[a,b#]**

[a]Department of Molecular and Cell Biology, University of California, Berkeley, California, USA

[b]Howard Hughes Medical Institute, University of California, Berkeley, California, USA

#Address correspondence to Nicole King, nking@berkeley.edu.

*Present address: Department of Microbiology, University of Texas Southwestern Medical Center, Dallas, Texas, USA

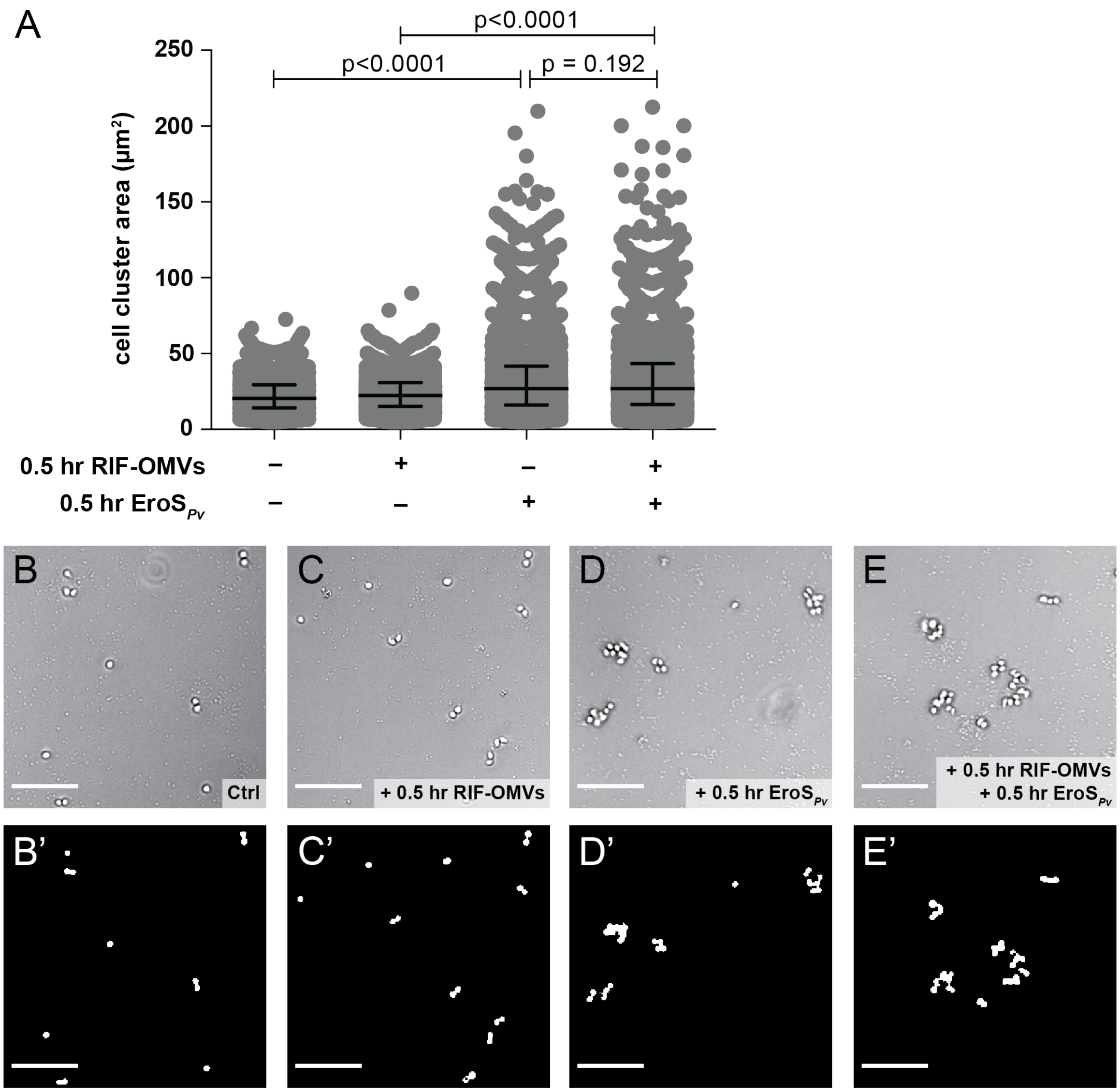


**Figure S1: RIF-OMVs have no effect on EroS$_{Pv}$-induced swarming**

(A) Solitary cells from SrEpac co-treated with a 1:1,000 dilution of RIF-OMVs and 0.05 U/mL (0.2-1 μg/mL, ~2-8 nM) EroS$_{Pv}$ formed swarms, quantifiable by an increase in cell cluster area (median = 27.0 μm$^2$, interquartile range = 16.5-43.5 μm$^2$) compared to cells treated with RIF-OMVs and BSA (carrier control) (median = 22.4 μm$^2$, interquartile range = 15.2-30.8 μm$^2$). There was no significant difference in swarm size between cells co-treated with RIF-OMVs and EroS$_{Pv}$ and cells treated

with $EroS_{Pv}$ alone (median = 27.0 $\mu m^2$, interquartile range = 16.0-41.8 $\mu m^2$) (Kolmogorov-Smirnov test). A minimum of 2730 cell cluster areas from 3 biological replicates were included in the scatter plot for each condition. (B-E') Sample images used for quantification in (A). Following the approach of (18), raw images in (B-E) were converted to binary images (B'-E') to measure cell cluster size (Materials and Methods). Scale bars = 50 µm. (B) *S. rosetta* cells from SrEpac treated with BSA (carrier control) remained solitary. (C) *S. rosetta* cells from SrEpac treated with a 1:1,000 dilution of RIF-OMVs and BSA (carrier control) for 0.5 hours remained solitary. (D) *S. rosetta* cells from SrEpac treated with 0.05 U/mL (0.2-1 µg/mL, ~2-8 nM) $EroS_{Pv}$ for 0.5 hours formed visible swarms. (E) *S. rosetta* cells from SrEpac co-treated with a 1:1,000 dilution of RIF-OMVs and 0.05 U/mL (0.2-1 µg/mL, ~2-8 nM) $EroS_{Pv}$ for 0.5 hours formed visible swarms.

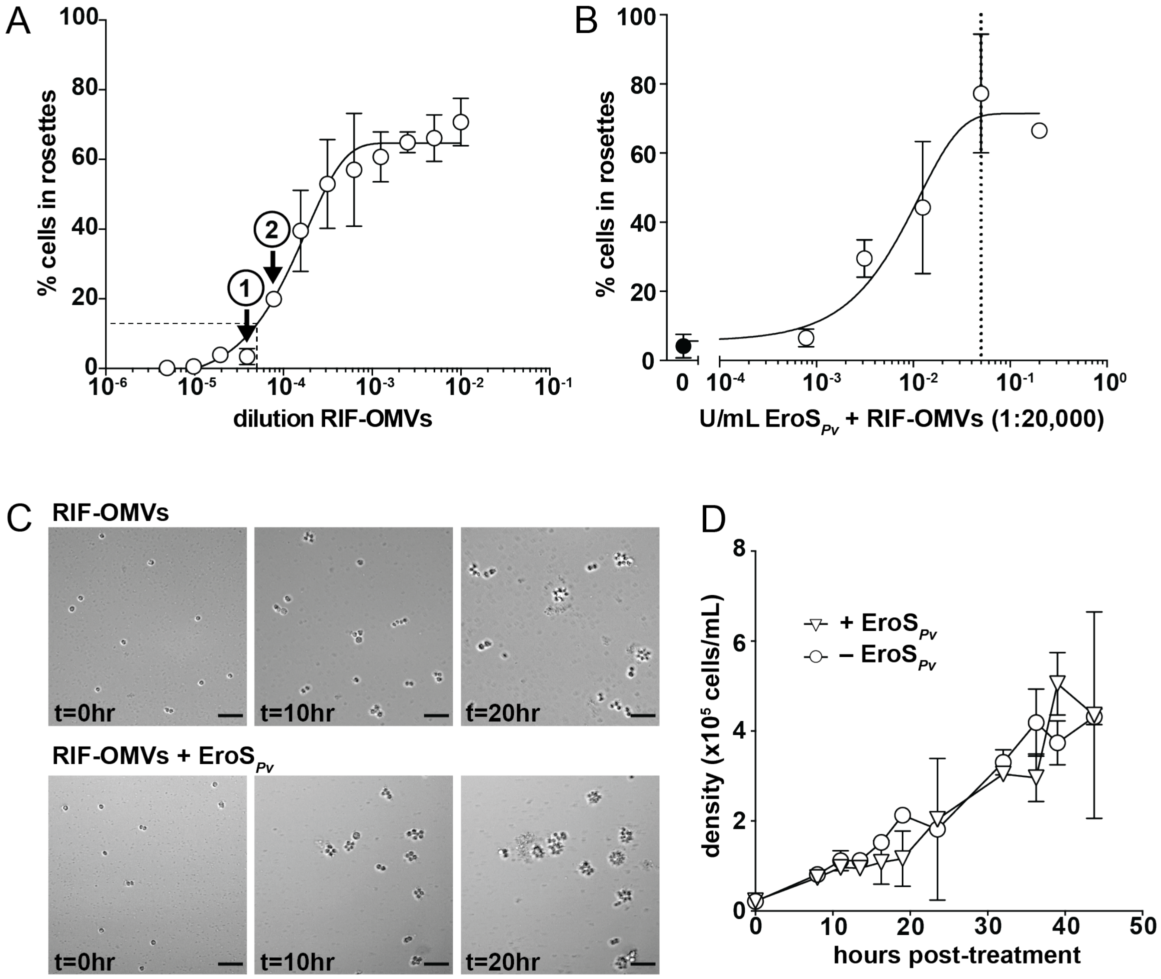


**Figure S2: EroS$_{Pv}$ enhances rosette development, but not cell proliferation, in a sensitized rosette induction assay**

(A) Serial dilution of RIF-OMVs can be used to induce a low percentage of cells in rosettes. SrEpac treated with a 1:25,600 dilution of RIF-OMVs resulted in 3.4±2.3 (mean ± S.D. of 3 biological replicates) *S. rosetta* cells in rosettes (arrow marked (**1**)), while a 1:12,800 dilution of RIF-OMVs resulted in 19.9±1.7 (mean ± S.D. of 3 biological replicates) *S. rosetta* cells in rosettes (arrow marked (**2**)). An intermediate dilution of 1:20,000 was used for the sensitized rosette induction assay (dashed

lines). (B) Rosette-enhancing activity correlated with EroS$_{Pv}$ concentration. SrEpac treated with a 1:20,000 dilution of RIF-OMVs and BSA (carrier control) (black circle) contained more *S. rosetta* cells in rosettes upon the addition of increasing concentrations of EroS$_{Pv}$ (white circles). Dotted line indicates concentration of EroS$_{Pv}$ (0.05 U/mL (0.2-1 μg/mL, ~2-8 nM)) used for subsequent assays. Mean ± S.D. of 3 biological replicates plotted. (C) Time-lapse imaging showed an increase in both the number of rosettes and the number of cells per rosette after co-treatment with a 1:20,000 dilution of RIF-OMVs and 0.05 U/mL (0.2-1 μg/mL, ~2-8 nM) EroS$_{Pv}$ (bottom) compared to RIF-OMVs and BSA (carrier control) (top). Scale bars = 100 μm. (D) *S. rosetta* cells treated with a 1:20,000 dilution of RIF-OMVs and BSA (carrier control) (circles) or co-treated with RIF-OMVs and 0.05 U/mL (0.2-1 μg/mL, ~2-8 nM) EroS$_{Pv}$ (triangles) grew at the same rate. Mean density ± S.D. of 3 biological replicates plotted.
